# Access to care, nativity and disease management among Latinos with diabetes in a safety-net healthcare setting

**DOI:** 10.3934/publichealth.2019.4.488

**Published:** 2019-11-18

**Authors:** Elizabeth Burner, Sophie Terp, Chun Nok Lam, Emily Neill, Michael Menchine, Sanjay Arora

**Affiliations:** 1 University of Southern California, Keck School of Medicine, LAC+USC Medical Center, Los Angeles, USA; 2 University of California San Francisco, San Francisco, USA

**Keywords:** Hispanic Americans, emigrants and immigrants, diabetes mellitus, glycosylated hemoglobin, health services accessibility

## Abstract

**Introduction:**

Latinos in the U.S. are disproportionately affected by diabetes and its complications. The role of access to care and nativity in diabetes management are important areas of research, as these findings can help direct tailored interventions.

**Methods:**

We examined associations between access to care, acculturation and glycemic control among Latino patients with diabetes seen in a safety net emergency department. We used regression models to estimate the individual predictors' associations with glycemic control and then estimated adjusted associations by controlling for all relevant predictors. We tested for a moderating role of nativity in the associations between access to care and glycemic control.

**Results:**

In unadjusted analysis, we found the most significant predictors of glycemic control to be access to primary care (β = −0.89, p = 0.011), capacity for self-monitoring glucose (β = −0.68, p = 0.022), mental health comorbidities (β = 0.95, p = 0.013), male gender (β = −0.49, p = 0.091) and nativity (β = −0.81, p = 0.034). In adjusted analysis, nativity was no longer a significant predictor of glycemic control (β = −0.32, p = 0.541). Nativity did not significantly moderate the association of access to care and glycemic control.

**Conclusions:**

Our findings show a direct association between access to care and glycemic control among low-income Latinos seeking care in the emergency department. This supports concerns that many researchers, clinicians and policy analysts have expressed regarding access to care for immigrants. The importance of primary care and access to supplies to perform self-management in achieving glycemic control and reducing risk of complications indicate that ensuring access to quality care is critical to the health of this vulnerable group.

## 1. Introduction

Latinos suffer from diabetes and related complications at disproportionate rates compared to non-Hispanic Whites in the United States (U.S.) [1]–[3]. Multiple factors including access to primary care, environmental challenges to physical activity and nutrition, and language barriers likely contribute to the notably higher rates of diabetes and complications among U.S. Latinos [4]–[9]. However, important subsets of the Latino population with diabetes are currently underrepresented in the scientific literature [10], especially those who rely on safety-net care settings such as emergency departments (EDs).

While patients with chronic diseases such as diabetes are best served in primary care settings, EDs play a large part in the care of patients with diabetes. The role of the ED may be expanding especially for those patients with diabetes who lack access to primary care; EDs have been proposed as feasible venues to screen for undiagnosed diabetes, particularly for Latino patients [11]–[13]. Patients with diabetes who seek care in the ED have exhibited worse disease management [14], and are need of interventions to prevent complications of diabetes. While ED-based interventions for improving diabetes management have been tested successfully [15],[16], we still do not know if these interventions should focus on improving access to care, mitigating social determinants of health or modifying individual factors. Understanding this may be particularly important among foreign-born patients with diabetes, as lack of access to care and sufficient social resources may result in further threats to disease management and good health outcomes.

Access to healthcare is a critical component of diabetes management. Patients with diabetes require regular access to healthcare for routine medication adjustments by providers, laboratory monitoring for treatment goals and side effects, encouragement to continue to make healthy behavior change, as well as yearly ophthalmic and dental exams to achieve optimal self-care [17]. Additionally, they must have access to a glucometer to self-monitor blood glucose and sufficient resources to purchase their prescription medications to be able to effectively manage their chronic disease [18]. All of these factors related to access to care impact the average blood glucose of a patient. The most commonly used clinical measure of chronic disease management in patients with diabetes is A1C (percent glycosylated hemoglobin of the total hemoglobin measurement) which measures a patient's average blood glucose over the prior three months. A1C is associated long-term health outcomes including heart attack, stroke, kidney failure and blindness. An increase of 1% of HbA1c above the target of 7.5% is associated with a 4% increased relative risk of all-cause mortality [19]. Patients with inadequate access to care are more likely to suffer such long-term complications of uncontrolled diabetes [20]. Latinos are also more likely to lack medical insurance, which supplies necessary equipment to self-monitor blood glucose and perform adequate self-care [21]. While in aggregate, Latinos with diabetes are affected by barriers to accessing to care, these barriers to access to care may be a greater issue for foreign-born patients. Foreign-born patients are more likely to have low-English proficiency and are less likely to have medical insurance, which are both associated with worse chronic disease management and health outcomes [22],[23].

To better design interventions to combat disparities in diabetes management and glycemic control, we need to better understand how immigration and language barriers interact with access to care to impact chronic control of diabetes and risk of diabetic complications. In this cross-sectional study of Latino patients with diabetes seeking care in an urban ED, we examine the moderating effect of immigration on the relationship of access to healthcare with diabetes control as measured by glycosylated (HbA1C).

## 2. Methods

This prospective, cross-sectional study was conducted in the ED at Los Angeles County + University of Southern California (LAC+USC) Medical Center in two waves in 2010 and 2013. LAC+USC is a large, urban, public medical center with more than 170,000 visits annually. The population served is predominantly low-income, Spanish-speaking and Latino. All procedures were in accordance with the ethical standards of the responsible committee on human experimentation (institutional and national) and with the Helsinki Declaration of 1975, as revised in 2000. Informed consent was obtained from all participants included in the study. Patients were eligible for participation if they had diabetes (type 1 or 2), were over 18 years of age and could provide informed consent. Patients were excluded if they were 1) critically ill; 2) in police custody; 3) suffering from acute psychosis or 4) otherwise unable to give informed consent. During standard business hours, consecutive patients were approached if they had a diagnosis of diabetes in the electronic medical record, regardless of reason for visit that day. Patients were interviewed in English or Spanish, depending on their language preference. Patients were queried on gender, ethnicity, educational attainment, income, language preference, country of birth, healthcare utilization, co-morbidities, access to care and ownership of a glucometer to monitor their diabetes at home. Weight, height and glycosylated hemoglobin (HbA1c) were collected at the time of the interview. In the second wave of interviews, patients were also queried on their documentation status (irregular or documented).

### 2.1. Measures

*Glycemic Control*: To measure diabetes management and glycemic control, we collected HbA1c at the time of the interview using the Afinion™ AS 100 HbA1c point of care testing machine, which has previously been demonstrated to have excellent correlation with serum values [24].

#### 2.1.1. Nativity

*Nativity*: Patients were categorized as foreign-born if they reported being born outside the U.S. to parents who also had not been born in the U.S.

#### 2.1.2. Demographics

*Age*: Age from the clinical chart.

*Gender*: Patient's self-reported gender.

*Education Level*: Patients reported if they had completed less than high school, high school, some college or trade school, or completed college or trade school. Due to low responses in the two higher categories, education level was collapsed to complete less than high school education or completed high school or obtained a more advanced degree.

#### 2.1.3. Acculturation

*English Proficiency*: Patients that spoke a language other than English were asked to answer U.S. census-style question regarding their English proficiency. It they spoke English less than “Very Well” they were categorized as low English proficiency. We used this as a brief marker of acculturation.

#### 2.1.4. Comorbidities

*Mental Health Condition*: Presence of a mental health condition was determined by the patient response to a checklist of comorbidities, which included depression, anxiety, schizophrenia or an “other” write-in response that was reviewed by two senior clinicians (e.g. “bipolar” and “manic-depressive”).

*Obesity*: Body Mass Index (BMI) was calculated using height and weight collected at time of survey. A BMI of 30 or greater was categorized as obese.

#### 2.1.5. Access to care

*Insurance*: Patients reported whether they had no insurance, participated in a low-income insurance program such as Medicaid, or had non-means tested insurance such as Medicare, private insurance or employer-sponsored insurance.

*Primary Care*: Patients reported if they had seen their primary care provider in the previous 12 months.

*Capacity to Self-Monitor Blood Glucose*: We asked patients if they owned a glucometer, as a marker of patients having sufficient access to health care resources to own a glucometer.

#### 2.1.6. Immigration documentation status

For the second wave only, we asked a series of questions to determine if foreign-born patients were irregular immigrants. We asked if they were 1) naturalized citizens; 2) legal permanent residents with green cards or 3) granted Visas. Those who preferred not to answer and or responded application pending were categorized as irregular immigrants, a categorization used by the Pew Hispanic Center and the American Community Survey [25].

### 2.2. Statistical analysis

For this study, only patients that self-identified as Latino or Hispanic were retained for data analysis. For initial univariate analysis, we compared U.S.-born to foreign-born Latinos across all variables described above, using t-tests or chi-squared tests as appropriate. All analysis was performed with Stata version 13.1 [26]. Patients who did not complete the survey or consent to blood draw from HbA1c measurement were excluded from analysis. We initially examined the relationship between the individual variables and glycemic control via linear regression. We then adjusted these estimates with a multivariate model that included all variables from the univariate analysis. Due to concern for multi-colinearity, we checked variable inflation factors prior to running the adjusted model. For patients who had data available on documentation status, we conducted a second adjusted analysis incorporating documentation status. Additionally, we checked for interaction between nativity and each of the access to care predictor variables on the outcomes of glycemic control. Lastly, we performed a power calculation to determine if the study was adequately powered to identify a difference in HbA1c while including all variables of interest using the STATA *power* command with the *rsquared* option.

## 3. Results

361 Latino patients with diabetes were identified and enrolled: 300 foreign-born and 61 U.S.-born Latinos. A total of 313 patients completed the survey and provided a blood sample to measure HbA1c, and were included in the multivariate analysis. In total, respondents were predominantly foreign-born (83%), had low English proficiency (70% spoke English less than very well) and limited educational attainment (61% with less than high school education). Nearly half were obese and one in six reported history of a mental health condition. While over half of patients had no medical insurance, over three quarters had received primary care in the last year. More than half had a glucometer in their home. A summary of patient characteristics is included in Table 1.

### 3.1. Power calculation

The sample size of 313 patients with complete data gave us 98% power to calculate a difference in HbA1c of 0.11 using 13 covariates.

### 3.2. Unadjusted analysis

Foreign-born patients differed from U.S.-born patients in several areas (Table 1). Foreign-born patients were more likely to be older and to have lower educational attainment than their U.S.-born counterparts (p < 0.001 for each). They were more often Spanish-speaking with low English proficiency. Foreign-born patients exhibited less obesity than U.S.-born patients (p = 0.003). While they were less likely to have insurance than US-born patients, foreign-born patients were more likely to have received primary care in the last year and to own a glucometer. Foreign-born patients trended to better glycemic control, with a mean HbA1c of 8.6% of total hemoglobin compared to 9.4% among U.S.-born patients (p = 0.059).

**Table 1.** Demographic characteristics among study participants (n = 313).

	Total		Foreign-Born		US-Born		p-value
	n = 313	% (or SD)	n = 258	%(or SD)	n = 55	%(or SD)	
Age (mean, SD)	53	11.80	54	11.11	47	13.57	< 0.001
Male	141	45.05	111	43%	30	55%	0.119
High School Education or Higher	121	38%	77	30%	44	80%	< 0.001
High English Proficiency	95	30%	42	16%	53	96%	< 0.001
HbA1c (mean, SD)	8.77	2.65	8.63	2.53	9.44	2.66	0.034
% HbA1C > 8	164	52%	129	50%	35	63%	0.066
Mental Health Condition	54	17%	40	16%	14	25%	0.076
Obesity	153	49%	117	45%	36	65%	0.007
Insurance Coverage							
None	195	62%	165	64%	30	55%	0.191
Non-means Tested Insurance (i.e. Medicare, private)	47	15%	31	12%	16	29%	0.001
Low-income Insurance Program (i.e. Medi-CAL)	71	23%	62	24%	9	16%	0.218
Primary Care (Visit in last year)	244	78%	204	79%	40	73%	0.303
Capacity for Self-Monitoring Glucose	187	60%	158	61%	29	53%	0.242

### 3.3. Unadjusted and adjusted analysis

As all of our tests for co-linearity had variable inflation factors of less than 2.5, we included all initial variables in the multivariate model. No interaction terms between nativity and individual variables were statistically significant.

### 3.4. Nativity

Glycemic control among immigrants was significantly better than U.S.-born Latinos in unadjusted analysis. Foreign-born patients exhibited HbA1c 0.81% lower than U.S.-born patients (p = 0.03). This magnitude decreased in adjusted analysis, and was no longer statistically significant (Table 2). Nativity did not have a significantly moderate the association between glycemic control and any of the other predictor variables.

**Table 2.** Coefficients of linear regression analyses on hba1c by selected patient characteristics (n = 313).

	Unadjusted Coefficients				Adjusted Coefficients			
	β	95%	CI	p-value	β	95%	CI	p-value
Nativity								
Foreign Born	−0.81	−1.55	−1.55	0.034	−0.32	−1.33	0.70	0.541
Demographic								
Age	−0.03	−0.05	−0.05	0.014	−0.02	−0.05	0.01	0.118
Male	−0.49	−1.06	−1.06	0.091	−0.78	−1.37	−0.20	0.009
High School Education or Higher	0.35	−0.24	−0.24	0.246	0.08	−0.59	0.76	0.809
Acculturation								
High English Proficiency	0.61	−0.01	−0.01	0.052	0.34	−0.51	1.18	0.434
Comorbidities								
Mental Health Condition	0.95	0.2	0.2	0.013	0.93	0.18	1.68	0.016
Obesity	0.04	−0.54	−0.54	0.904	−0.14	−0.72	0.44	0.640
Access to Care								
Insurance Coverage								
Non-means Tested Insurance (i.e. Medicare, private)	−0.21	−0.89	−0.89	0.542	−0.21	−0.91	0.50	0.561
Low-income Insurance Program (i.e. Medi-CAL)	−0.09	−0.89	−0.89	0.827	−0.23	−1.06	0.59	0.579
Primary Care (visit in last year)	−0.89	−1.58	−1.58	0.011	−0.82	−1.51	−0.12	0.021
Capacity for Self-Monitoring Glucose	−0.68	−1.26	−1.26	0.022	−0.56	−-1.14	0.02	0.057

### 3.5. Demographics

Several demographic factors had significant associations with glycemic control. Age had a significant negative correlation with HbA1c (better glycemic control), with decrease in HbA1c of 0.3% for every ten years of increased age (p = 0.01). The age benefit did not retain significance in adjusted analysis. Male gender also corresponded with a significantly lower HbA1c, with men averaging HbA1c 0.49% lower than their female counterparts, which remained significant in adjusted analysis (p = 0.009). Educational attainment, our marker of socioeconomic status, had no association with glycemic control, before and after controlling for all other variables.

### 3.6. English proficiency

Our acculturation proxy, high English proficiency, was marginally associated with worse glycemic control, however showed no significant association with glycemic control when controlling for other variables.

### 3.7. Comorbidities

Patients reporting a mental health conditions had worse glycemic control, with those patients with a mental health condition having a mean HbA1c 0.93% higher (p = 0.02) than those without mental health conditions when controlling for nativity, demographic predictors, acculturation, obesity and access to care. We found that obesity did not significantly correlate with glycemic control in unadjusted or adjusted analysis.

### 3.8. Access to care

Primary care was significantly associated with improved glycemic control in both analysis, with patients who reported a primary care visit in the prior 12 months exhibiting 0.89% lower HbA1c (exhibited better glycemic control) than those who did not report a primary care visit, adjusting for all other factors, p = 0.011. Similarly, having the capacity to self-monitor blood glucose was associated with a 0.68% (p = 0.02) decrease in HbA1c, however this relationship decreased slightly (to mean 0.56% lower) and became marginal when controlling for all other variables. Type of medical insurance was not significantly associated with glycemic control in adjusted or unadjusted analysis.

### 3.9. Documentation status

For the patients with complete data in the second wave (which included documentation status), we also constructed a multivariate analysis using the 11 variables in the adjusted model, adding documentation status. There was no association between documentation status and glycemic control in either the univariate analysis or final multivariate model.

## 4. Discussion

In this study of Latino patients with diabetes in a safety net ED, we explored factors related to access to care associated with glycemic control, with a focus on the moderating effect of nativity. While there was not a significant moderating role of nativity on access to care, we found that the most significant predictors of glycemic control were gender, mental health comorbidities, access to primary care and capacity to self-monitor blood glucose. These findings highlight the multifactorial nature of chronic disease management, and the challenges that this population of primarily foreign-born Latinos experience in managing their diabetes.

Access to care for resource-poor foreign-born Latinos contributes significantly to management of diabetes. Patients with better access to care are better able to manage their diabetes, as shown by improved HbA1c, when other factors are controlled for. Access to care has previously been shown to be influenced by racial and ethnic differences in insurance status [27]. As having a medical home is important for diabetes management for foreign-born Latinos [28],[29], this study provides further evidence that restricting access to primary and preventive care for foreign-born Latinos may result in poor health outcomes, particularly for those with chronic diseases. While in previous work, foreign-born patients have been less likely to have a regular access to care [30], our study presents a unique situation to study access to care. LAC+USC is a large safety net hospital that is part of a county-wide system providing care to many foreign-born residents. Additionally, as the metropolitan area to the second largest population of Latinos in the world (behind México City) [31] with a high proportion of immigrants, LAC+USC presents a unique opportunity to separate language and cultural barriers to care from other specific access factors. For these patients, adequate access to primary care and physical supplies necessary for self-management were the most significant factors associated with improved glycemic control. Additionally, interaction terms for access to care and nativity were not significant, suggesting that foreign-born patients benefit from primary care to the same degree as U.S.-born patients and the investment in their health would have similar benefits.

This study also addresses role of gender in glycemic control for Latino patients. Our male patients showed better glycemic control then our female patients, when controlling for other factors. This gender difference is consistent with prior literature highlighting differences in diabetes management between men and women. In non-Hispanic whites, male gender is associated with better diet and physical activity and has a protective effect on glycemic control, while women more often report better social support, which has separately been associated with better glycemic control [32]–[35]. Our findings are consistent with prior studies, which have shown gender differences in diabetes management for Latinos as well [36]–[38]. Given the highly gendered roles associated with health in some Latino communities [39], it is likely that men and women experience the challenges of diabetes management differently.

The patients with a mental health condition trended toward worse glycemic control than patients without a mental health condition. Patients with diabetes are known to have higher rates of mental health comorbidities, in particular depression and anxiety [40],[41]. While U.S. Latinos have lower rates of mental health conditions than the U.S. population at large, particularly among foreign-born Latinos, mental health comorbidities are associated with higher rates of death among Latinos [42]–[44]. Mental health conditions negatively impact diabetes management and self-care behaviors in both non-Hispanic whites and Latinos [44]–[46]. Our findings support the role of mental health in glycemic control for Latinos and the importance of recognizing and treating these comorbidities that can have a different presentation among Latinos than other segments of the U.S. population.

We did not find an association between acculturation (as measured by English proficiency) and glycemic control, in contrast to work by other groups [23],[47]–[50]. Schwartz et al define acculturation as the changes that take place as a result of contact with culturally dissimilar people, groups, and social influences [51]. Acculturation is multi-dimensional, and language alone does not fully encompass acculturation. However, language preference has been used successfully in diabetes self-care research [52]. Acculturation and the “immigrant paradox” are believed to drive findings of better health in immigrant communities despite generally lower socio-economic status, particularly for Latinos [51]. Residence in immigrant enclaves may explain the lower rates of other chronic diseases for foreign-born Latinos compared to U.S.-born Latinos, but have mixed findings in diabetes [53],[54]. Others suggest that unexpectedly low reported rates of diabetes may reflect under diagnosis of diabetes among immigrants with poor access to care rather than true low rates of disease. Previous work identified a link between acculturation and poor diet, physical activity levels and health outcomes for patients with diabetes [55],[56]. Others have not found this association between acculturation and poor health outcomes [23],[57]. We found no relationship between acculturation and glycemic control, potentially due to the study setting in a largely Spanish-speaking and foreign-born neighborhood, potentially mitigating the effect seen in other settings.

While addressing a population that is underrepresented in the literature, there are a number of limitations to this study. The patients represented are not a random sample of Latinos, and in fact, foreign-born Latinos are over-represented compared to the national Latino population, which limits the generalizability of our findings. However, LAC+USC is a unique site to study foreign-born Latinos, as they represent a majority of the patients seen at the facility. Given the safety net function of this hospital, there was too little variation in income to include it in our models. However, given the very low income reported by our patients, we were able to study a group that is underrepresented in medical literature. While this is a cross-sectional study with a convenience sample, which limits our ability to investigate causation and the generalizability of our findings, the previously understudied patients represented in this study are an important group to highlight. An additional limitation of this study design is the reliance on self-report for many of our variables. We relied on patient self-report of diabetes to nurses and research assistants to approach subjects for inclusion, which could miss potentially eligible patients with known diabetes, or those with undiagnosed disease. However, previous work by our group showed excellent sensitivity and specificity to correctly identifying those patients who had previously been diagnosed with diabetes [58], so we are confident that we captured those who knew of their diagnosis. We also relied on self-report of mental health comorbidities, male gender, country of birth, nativity, documentation of immigration, language proficiency, education level, insurance status and access to care. The self-report of access to primary care is particularly subject to recall bias, as patients may not accurately recall their most recent primary care visit, or recognize their primary care visit as such. Additionally, they may inaccurately consider the ED to provide them with primary care. We also could not objectively assess the quality of primary care received by these patients, and our findings of the role of access to care must be viewed in this context. We relied on self-report of mental health conditions. Clinically testing for mental health conditions or other confirmatory methods for these self-report variables was not feasible given the logistical constraints of this ED based study, but would have strengthened this study's validity. As we were unable to perform clinical testing, we used a dichotomous presence or absence of mental illness to prevent bias from patient confusion regarding an exact diagnosis.

## 5. Conclusions

In this study with a focus on nativity and access to care among low-income Latinos, access to care was a significant predictor of good glycemic control, along with gender and mental health comorbidities. The importance of primary care and access to supplies to perform self-management in achieving good glycemic control and reducing risk of complications indicate that ensuring access to quality care is critical to the health of immigrants with diabetes.
